# Constructing Injectable Bone-Forming Units by Loading a Subtype of Osteoprogenitors on Decellularized Bone Matrix Powders for Bone Regeneration

**DOI:** 10.3389/fcell.2022.910819

**Published:** 2022-07-06

**Authors:** Yan Xu, Shaohang Yan, Can Chen, Bangbao Lu, Ruibo Zhao

**Affiliations:** ^1^ Department of Sports Medicine, Xiangya Hospital, Central South University, Changsha, China; ^2^ National Clinical Research Center for Geriatric Disorders, Xiangya Hospital, Central South University, Changsha, China; ^3^ Department of Orthopedics, Xiangya Hospital, Central South University, Changsha, China

**Keywords:** bone defect, stem cell subpopulation, decellularized bone matrix, bone tissue engineering, bone-forming unit

## Abstract

Bone defects resulting from trauma or tumor are one of the most challenging problems in clinical settings. Current tissue engineering (TE) strategies for managing bone defects are insufficient, owing to without using optimal osteoconductive material and seeding cells capable of superior osteogenic potential; thus their efficacy is instable. Herein, a novel TE strategy was developed for treating bone defects. First, the decellularized bone matrix (DBM) was manufactured into powders, and these DBM powders preserved the ultrastructural and compositional properties of native trabecular bone, are non-cytotoxic and low-immunogenic, and are capable of inducing the interacted stem cells differentiating into osteogenic lineage. Then, a subtype of osteoprogenitors was isolated from mouse long bones, and its high osteogenic potential was identified *in vitro*. After that, we constructed a “bone-forming unit” by seeding the special subtype of osteoprogenitors onto the DBM powders. *In vivo* performance of the “bone-forming units” was determined by injecting into the defect site of a mouse femoral epiphysis bone defect model. The results indicated that the “bone-forming unit” was capable of enhancing bone defect healing by regulating new bone formation and remodeling. Overall, the study establishes a protocol to construct a novel “bone-forming unit,” which may be an alternative strategy in future bone TE application.

## Introduction

Severe trauma, congenital disorders, bone tumor, or infectious diseases can cause severe damage to the bone structure and function, leading to large bone defects (Safdari et al., 2021). Insufficient blood supply, bone infection, and systemic diseases can adversely influence bone healing, resulting in delayed union or nonunion of the bone (Giannoudis et al., 2016; Zakhary and Thakker, 2017; Biggemann et al., 2018). Currently, autologous bone grafts, allografts, and tissue-engineered bone graft have become the main strategies for treating large bone defects (Safdari et al., 2021). Autologous bone graft from the patient’s ilium is the gold standard of bone defect treatment (Sen and Miclau, 2007). However, donor-site morbidity, high rates of infections, and graft size limitations render this strategy unsuitable in many cases (Junka et al., 2020). In addition, the use of allografts, mostly acquired from the cadaveric body, face the risk of graft rejection and pathogen transmission (Perry, 1999). Fortunately, the advancement in bone tissue engineering (TE) has enabled the development of grafts consisting of stem cells and osteoconductive materials, which are extensively studied and considered as a potential option that can replace autograft and allograft (Kim et al., 2017; Turnbull et al., 2018). However, until now, the optimal osteoconductive material and stem cells for bone TE are yet to be defined.

In the past years, developing osteoconductive materials have been extensively studied. Recently, the decellularized matrix has gained popularity in the TE field due to its low immunogenicity, high biocompatibility, good biodegradability, and high similarity to target tissue in morphology and ingredients (Cheng et al., 2014; Jakus et al., 2017; Chen et al., 2019). Additionally, the decellularized matrix can act as a suitable scaffold for cellular delivery, regulating the attached cells in proliferation and differentiation (Cheng et al., 2014; Jakus et al., 2017; Chen et al., 2019). Lee et al. (2014) decellularized bone tissue, and progenitor cells were seeded in an attempt to evaluate the potential of the decellularized bone matrix (DBM) for bone regeneration. This study showed that DBM has the property of osteogenic inducibility *in vitro* and osteoconduction *in vivo*. However, its application in bone TE is inadequate, owing to its low porosity and inconvenience for cell migration into the matrix to regenerate bone. In this regard, physically processing the DBM into powder is innovatively developed in this study, which can increase the surface area of the DBM for the attachment and interaction of target cells (Li et al., 2021). Moreover, this decellularized bone matrix powder (DBMP) is convenient for cells infiltrating into the matrix to regenerate bone. However, the use of DBM powder alone remains challenging in many aspects, such as insufficient cells to form bone tissue and difficulty in handling.

Toward these challenges, we introduced a strategy that constructs a “bone-forming unit” by loading novel osteoprogenitors on the DBMPs and delivers the bone-forming units into the defect site using an injectable fibrin glue (Shanghai RAAS; China) to enhance bone regeneration. Unlike traditional bone marrow mesenchymal stem cells (BMSCs) isolated by artificial plastic adhesion and being undefined and heterogeneous, these novel osteoprogenitors are freshly isolated by flow cytometry, which are purer and more representative of the endogenous cell types (Gulati et al., 2018). Moreover, this subtype of osteoprogenitors showed significantly better osteogenic differentiation ability with respect to the traditional BMSCs (Gulati et al., 2018). Furthermore, an injectable and commercial fibrin glue was used to deliver the DBMPs together with the attaching osteoprogenitors into the bone defect site, which would enable better handling in clinics. Currently, numerous literature reports indicated that fibrin glue loaded with stem cells is an effective strategy for treating bone defects (Noori et al., 2017; Ortiz et al., 2021). Fibrin glue is a natural polymer involved in the coagulation process, which allows for a uniform distribution of the DBMPs and the attaching osteoprogenitors filling the defect site (Noori et al., 2017). Moreover, it presents a porous structure that favors angiogenesis, extracellular matrix deposition, and cell–matrix interactions (Ortiz et al., 2021).

In this study, a novel TE strategy was developed for treating bone defects; a subtype of osteoprogenitors with high osteogenic potential was seeded on the DBMPs to construct a bone-forming unit, and then, the bone-forming units were delivered into the defect site using fibrin glue, by which we expected to preliminarily establish an approach that could generate bone-forming units for bone regeneration. Our study mainly consists of three parts: 1) a subtype of osteoprogenitor was isolated, and its high osteogenic potential was identified; 2) DBMPs were prepared and their osteogenic effects on osteoprogenitors were assessed *in vitro*; 3) bone-forming units were constructed by seeding the special osteoprogenitors on the DBMPs and then injecting into the mouse femoral epiphysis bone defect to determine the *in vivo* performance of the bone-forming units on bone regeneration. Overall, the injectable bone-forming units may be an alternative strategy in future bone TE application.

## Materials and Methods

### Isolation of Osteoprogenitors

According to the published literature, the novel osteoprogenitors were isolated (Gulati et al., 2018) and were named as B-cell lymphocyte-stimulating population (BLSP). Briefly, after dissection of the long bones from the mouse, these bones were dissociated into a single-cell suspension by type II collagenase digestion, following mechanical digestion with mortar and pestle. After that, we removed red blood cells by using ammonium-chloride-potassium (ACK) lysis buffer and TER119^+^ and CD45^+^ hematopoietic cells by magnetic-activated cell sorting (MACS); the BLSP was sorted out using a FACSAria cell sorter (BD Bioscience) with the sorting panel listed in Table 1.

**TABLE 1:** List of the sorting panel.

Marker	Conjugation
CD45	Alexa 700
Ter119	Alexa 700
Tie2	FITC
6C3	Percp/Cy5.5
CD90	APC/Cy7
ITGAV	BV421
CD105	PE/Cy7

### Evaluation of the Osteogenic Potential of Osteoprogenitors

In this study, BMSCs isolated with conventional protocol were selected as a control group. *In vitro* osteogenic potential of the sorted BLSP was evaluated with the methods described in previous literature reports (Breitbach et al., 2018). Briefly, the BMSCs (passage 3) or the BLSP were seeded at 3 × 10^4^ cells/well in a 24-well plate. To induce osteogenic differentiation, cells were cultured in an induction medium (0.1 μM dexamethasone, 500 μM ascorbic acid, and 10 mM β-glycerol phosphate in MEM alpha/10% FCS). The medium was changed every 3 days.

After 7 days induction, the expression of Runx2 in the two kinds of cells was evaluated by immunofluorescence staining to evaluate their osteogenic potential. Briefly, after being fixed in 4% (v/v) paraformaldehyde solution for 30 min, the cells were immersed in 0.1% Triton X‐100 (T8787, Sigma‐Aldrich), and then, nonspecific binding was blocked with 1% bovine serum albumin. Primary antibodies against Runx2 (ab192256, Abcam) were added to the surface of cells and incubated at 4°C overnight. After that, the corresponding secondary antibodies were combined for 30 min before DAPI staining. Images were captured using a confocal microscope (TCS‐SP8; Leica). In addition, the expression of osteogenic genes (Runx2 and Alp) was also evaluated using quantitative real‐time polymerase chain reaction analysis (qRT‐PCR). Briefly, the total RNA was extracted from the BMSCs or osteoprogenitors. The messenger RNA was reversely transcribed to complementary DNA by using the Super Quick RT MasterMix Kit (CW2391; Cwbiotech, Beijing, China). The mouse-specific primers for osteogenic (Runx2 and Alp) and housekeeping (β‐actin) genes were designed by Sango Biotech (Shanghai, China), which are shown in Table 2. The expression of the target gene was calculated using a LightCycler melting curve analysis and normalized with the housekeeping gene (GAPDH). After 21 days induction, the osteogenic potential of two kinds of cells was also evaluated by detecting the mineralized nodules using 2% alizarin red solution.

**TABLE 2:** Primer sequences used for qRT-PCR analysis.

Osteogenic gene	Primer sequence (5–3′)
Runx2	Forward CTG​CCA​CCT​CTG​ACT​TCT​GC
	Reverse GAT​GAA​ATG​CCT​GGG​AAC​TG
Alp	Forward TGA​CTA​CCA​CTC​GGG​TGA​ACC
	Reverse TGA​TAT​GCG​ATG​TCC​TTG​CAG
GAPDH	Forward AGG​TCG​GTG​TGA​ACG​GAT​TTG
	Reverse TGT​AGA​CCA​TGT​AGT​TGA​GGT​CA

### Preparation of DBMPs

The trabecular bone tissues were dissected from a pig’s spinal column at a local slaughterhouse. After that, the bone tissues were trimmed into blocks and decellularized with the following method. Briefly, bone tissues were immersed into sodium dodecyl sulfate (SDS; Sigma‐Aldrich Ltd.) dissolved in 0.1% Triton X‐100 with 2% concentration for 12 h under vigorous agitation at 4°C. After being washed with phosphate‐buffered saline (PBS) at 4°C three times (8 h per each time), they were digested in a nuclease solution (containing 500 U/ml DNase Type I and 1 mg/ml RNase) with agitation at 37°C for 12 h. After washing with PBS three times (8 h per each time) and lyophilizing in a vacuum freeze‐drier (FD8‐5T, SIM, Newark, NJ), the DBM was acquired. After that, the DBM was ground into powder.

### Evaluation of DBM Powders

After being fixed in 2.5% glutaraldehyde overnight at 4°C, the DBMPs and natural bone tissue (NBT) were dehydrated by a graded series of ethanol, and then sputter coated (IB‐5, EiKO, Tokyo, Japan) with gold. Then, the microstructure of the DBMPs was observed with SEM (Mira3 LMH, TESCAN, Czech Republic), and their contents of calcium (Ca) and phosphorus (P) were evaluated by energy dispersive spectrometry (EDS) analysis. Additionally, after the DBMPs and NBT were fixed in 4% (v/v) paraformaldehyde solution, they were decalcified and then embedded within paraffin, and sectioned with 5 μm thickness for hematoxylin and eosin (H&E), 4′,6-diamidino-2-phenylindole (DAPI), and Masson’s trichrome (MT) staining. H&E staining combined with DAPI was used for observing the elimination of cellular components in the DBMPs, while MT staining for observing the preservation of collagen in the DBMPs.

### Cytocompatibility of DBMPs

DBMPs were sterilized and immersed in DMEM/F‐12 overnight and then put on the bottom of the 24‐well plate (Corning) using polylysine (Corning). A total of 10^3^ BMSCs were, respectively, seeded onto DBMPs or tissue culture polystyrenes (TCPS) (as control). After 3-day incubation, the live cells or the dead cells on the DBMPs or TCPS (*n* = 4, each group) were, respectively, stained with calcein-AM (green) or EthD-1 (red), according to the instrument of the Live/Dead Assay kit (40747ES76, YEASEN, Shanghai, China). Meanwhile, after 1-, 3-, 5-, 7-, 9-, and 11-day culturing with complete medium, BMSC proliferation on the DBMPs or TCPS was quantified by using Cell Counting Kit‐8 (70-CCK8100, Multi Sciences, China).

### Immunogenicity of DBMPs

To determine the immunogenicity of DBMPs, we used 24-well Transwell inserts with 8-um pore size filters (Costar 3422, Corning, United States) (*n* = 3). Concisely, RAW 264.7 cells at a density of 5.0 × 10^4^ cells/well were seeded in the lower compartment with complete medium. The complete medium only was added to the upper compartment as a negative control (TCP), while the positive control was a complete medium containing 10 ug/ml lipopolysaccharide (LPS). The DBMP group was the complete medium containing DBMPs. After 7 days of culture, we measured the levels of TNF-a, IL-6, and IL-1β in the culture supernatants collected using ELISA kits (Multi Sciences, China).

### Osteogenic Inducibility of DBMPs

DBMPs were sterilized and immersed in DMEM/F‐12 overnight and then put on the bottom of the 24‐well plate (Corning) using polylysine (Corning). A total of 10^3^ BMSCs were, respectively, seeded onto DBMPs or TCPS (as control) and then cultured in an osteogenic induction medium (0.1 μM dexamethasone, 500 μM ascorbic acid, and 10 mM β-glycerol phosphate in MEM alpha/10% FCS). The medium was changed every 3 days.

After 7 days induction, the expression of Runx2 protein in the BMSCs cultured on DBMPs or TCPS was evaluated by immunofluorescence staining using primary antibodies against Runx2 (ab192256, Abcam). Meanwhile, the osteogenic genes (Runx2 and Alp) in the BMSCs cultured on DBMPs or TCPS were evaluated with qRT‐PCR. After 21 days induction, alizarin red staining was used to detect the osteogenic differentiation of the BMSCs cultured on DBMPs or TCPS.

### Construction of the Bone-forming Unit

In this study, the BLSP attached on a DBMP was named as the “bone-forming unit.” For the preparation of bone-forming units, mouse BLSP was used after immediately isolating from the mouse bone. Briefly, the cell suspension containing 5 × 10^6^ BMSCs or BLSP was seeded on the DBMPs (5 g) and then cultured within an ultra-low attachment plate (Corning, United States). After 1 day culture, the suspension containing bone-forming units was collected by centrifugation. The acquired precipitates immobilized in fibrin glue (Shanghai RAAS; China) were uniformly injected into the bone defect site. All procedures used for cell and animal experiments were performed in a sterile environment.

### Surgery and Treatment

A total of 88 mice were randomly divided into four experimental groups: the CTL group, DBMP group, BMSCs@DBMP group, and BLSP@DBMP group. A 0.6-mm femoral cylindrical defect in mice was selected as a critical-sized defect. The critical-sized bone defect in the CTL group, DBMP group, BMSCs@DBMP group, and BLSP@DBMP group was filled with fibrin glue, fibrin glue loading DBMPs, fibrin glue loading BMSCs@DBMPs, and fibrin glue loading BLSP@DBMPs, respectively. At 14 or 28 days postoperatively, all of the defected mice were euthanized, and the defected femur was dissected and fixed in 10 wt% neutral phosphate-buffered formalin solution to assess bone regeneration.

### Cells Labeling and Tracking

To track the fate and location of implanted cells (BLSPs or BMSCs), these BLSPs or BMSCs at the DBMPs were pre-stained with Dil stains (40757ES25, YEASEN, China). A non-invasive tracking system (IVIS Spectrum, PerkinElmer, United States) was used to image the Dil intensity and distribution on the mouse knee shoulder at the time point of injecting the cells as well as 3 or 7 days post-operation.

### Micro-CT Scanning

Trabecular bone architecture of the defected area was determined by Micro-CT (410 Versa, ZEISS, Solms, Germany) with a 0.5 μm isotropic voxel resolution under 40 kV voltage. The central 0.6-mm diameter and external 3-mm length region of the defect was defined as the volume of interest (VOI) to analyze the bone formation at the defect site. Trabecular bone volume per tissue volume (BV/TV) and trabecular thickness (Tb. Th) of VOI were measured.

### Histological Examination

After Micro-CT scanning, some femur specimens were decalcified and stained with H&E and MT for histopathological examination. The specimens were then dehydrated, embedded in paraffin, and then sliced into 5-μm sections. Subsequently, the section was stained with H&E and MT, following deparaffinization, and the images were captured by using an optical light microscope (Olympus, Japan). H&E-stained images were captured to calculate the percentage of the new bone area in the defect regions using the following formula: new bone formation area (%) = (new bone formation area/bone defect area) ×100%.

In addition, immunohistochemistry was performed to detect the expression of osteocalcin (Ocn) in the defect site at postoperative day 14. Sections were fixed, and the primary antibodies (rabbit polyclonal to osteocalcin, ab93876, Abcam) were diluted to the optimal concentration (1:100). The sections were then stained with primary anti-antibodies and visualized with a secondary antibody (Goat Anti-Rabbit IgG H&L, ab6721, Abcam). The immunohistochemistry images were observed using a light microscope (BX41, Olympus, Japan).

### Statistical Analysis

All data were described as mean ± standard deviation (mean ± SD). Analysis was performed with SPSS software (version 19; SPSS Inc., Chicago, IL, United States). A one-way analysis of variance (ANOVA) and *post hoc* Tukey test were carried out for comparisons between different groups. Statistical significance was set as *p* < 0.05.

## Results

### BLSP Sorting and Evaluation

**FIGURE 1:**
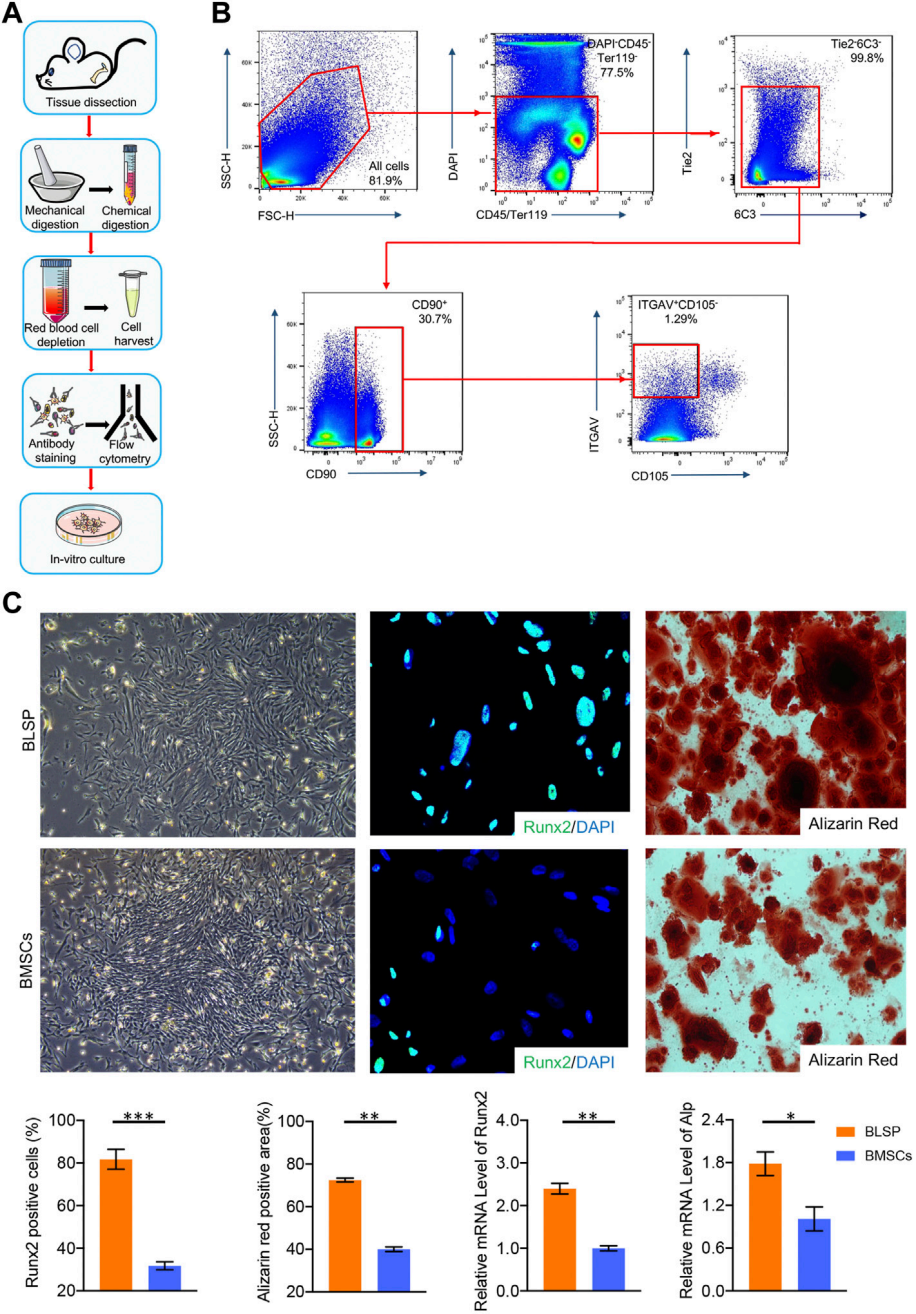
**(A)** Protocol for BLSP isolation, including the isolation of the mouse skeleton by tissue dissection, dissociation of the skeleton into a single-cell suspension by mechanical and chemical digestion, depletion of red blood cells with ACK lysis buffer, antibody staining of the BLSP, and sorting of the BLSP on a flow cytometer, BLSP culture. **(B)** Gating strategy used to sort the BLSP. Representative FACS plots with the percentage of the parent gate for BLSP. **(C)** Colony formation ability and osteogenic potential between BLSP and BMSCs. *n* = 4 for each group. Data are showed as means ± standard deviation (*p < 0.05, **p < 0.01, and ***p < 0.001).

According to the sorting procedures (Figure 1A), seven surface markers were used together to sort a special osteoprogenitor by a subsequent flow-cytometry-activated cell sorting (FACS) gating scheme, which was also named as BLSP (CD45^−^TER119^−^TIE2^−^ITGAV^+^THY1^+^6C3^−^CD105^−^) **(** Figure 1B **)**. Similar to the BMSCs isolated with conventional method, the isolated BLSP also displayed fibroblast-like morphology and still have colony-forming ability **(** Figure 1C **)**.

As shown in Figure 1C, both BLSP and BMSCs also showed osteogenic potential. However, in contrast to the BMSCs, much stronger osteogenic potential was observed in the BLSP. First, the positive staining area of alizarin red was significantly greater in the BLSP than in the BMSCs. Meanwhile, more BLSP expressed the Runx2 protein in comparison with the BMSCs. In addition, evaluation of the osteogenic gene expression using qRT-PCR further confirmed that the expression levels of Runx2 and Alp were significantly higher in the BLSP than the gene expression in the BMSCs. These results indicate that the BLSP possessed stronger osteogenic potential than BMSCs.

### Appearance and Morphology of DBMPs

**FIGURE 2:**
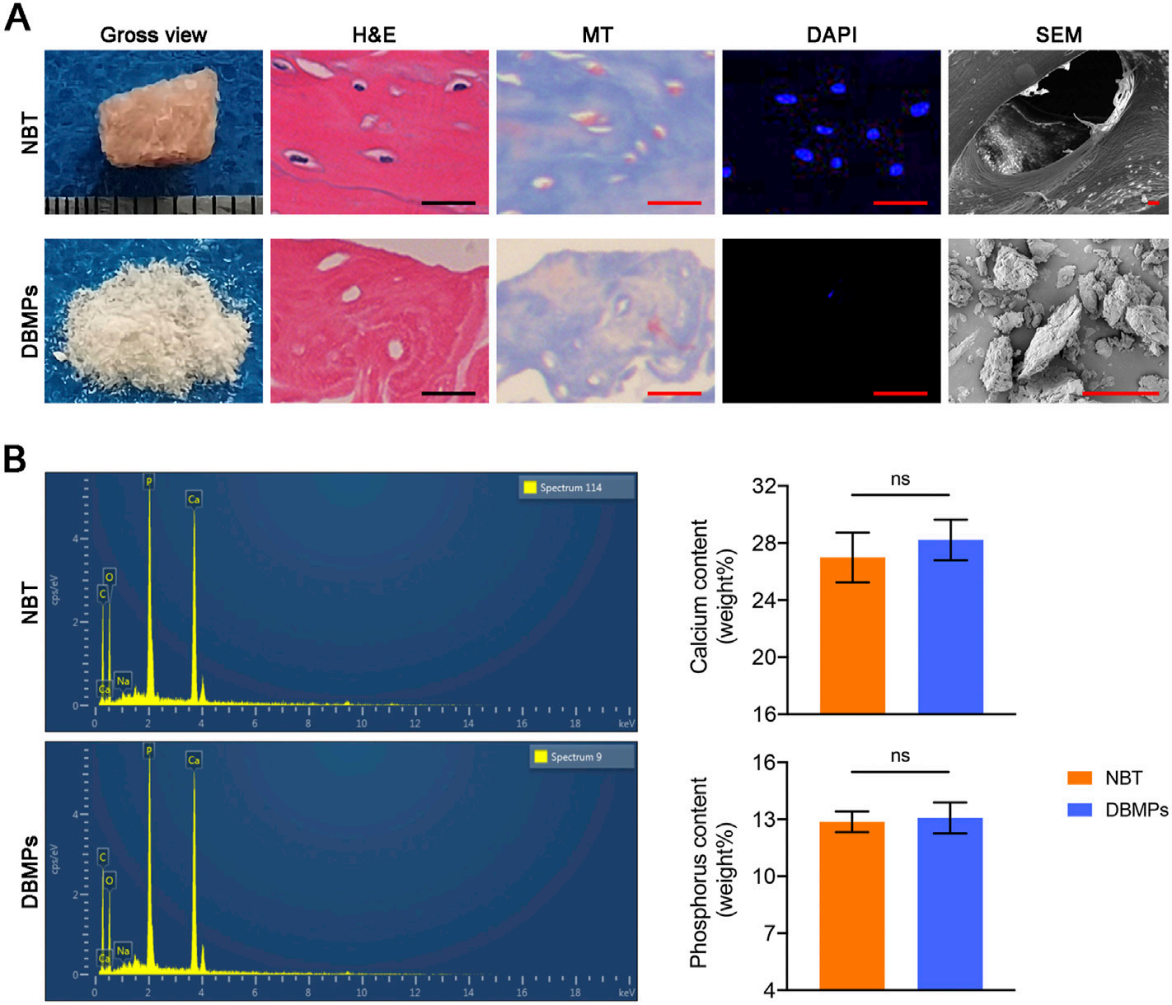
**(A)** Gross observation, H&E staining, MT staining, DAPI staining, SEM of the native bone tissue (NBT), and decellularized bone matrix powders (DBMPs). Bar = 10 μm. **(B)** Contents of calcium (Ca) and phosphorus (P) in the DBMPs and NBT evaluated by EDX. *n* = 4 for each group. Data are showed as means ± standard deviation (*p < 0.05, **p < 0.01, and ***p < 0.001).

The DBMPs were white in color with a diameter of about 1–8 μm (Figure 2A). Histologically, the DBMPs showed no cellular content in the H&E- and DAPI-stained images (Figure 2A). MT staining determined that the collagen content in the DBMPs presented a similar pattern with normal bone (Figure 2A). SEM images determined that the DBMPs preserved the ultrastructure of normal bone (Figure 2A). EDS analysis showed a similar content of calcium (Ca) and phosphorus (P) in the DBMPs and normal bone (Figure 2B).

### Cytocompatibility of DBMPs

**FIGURE 3:**
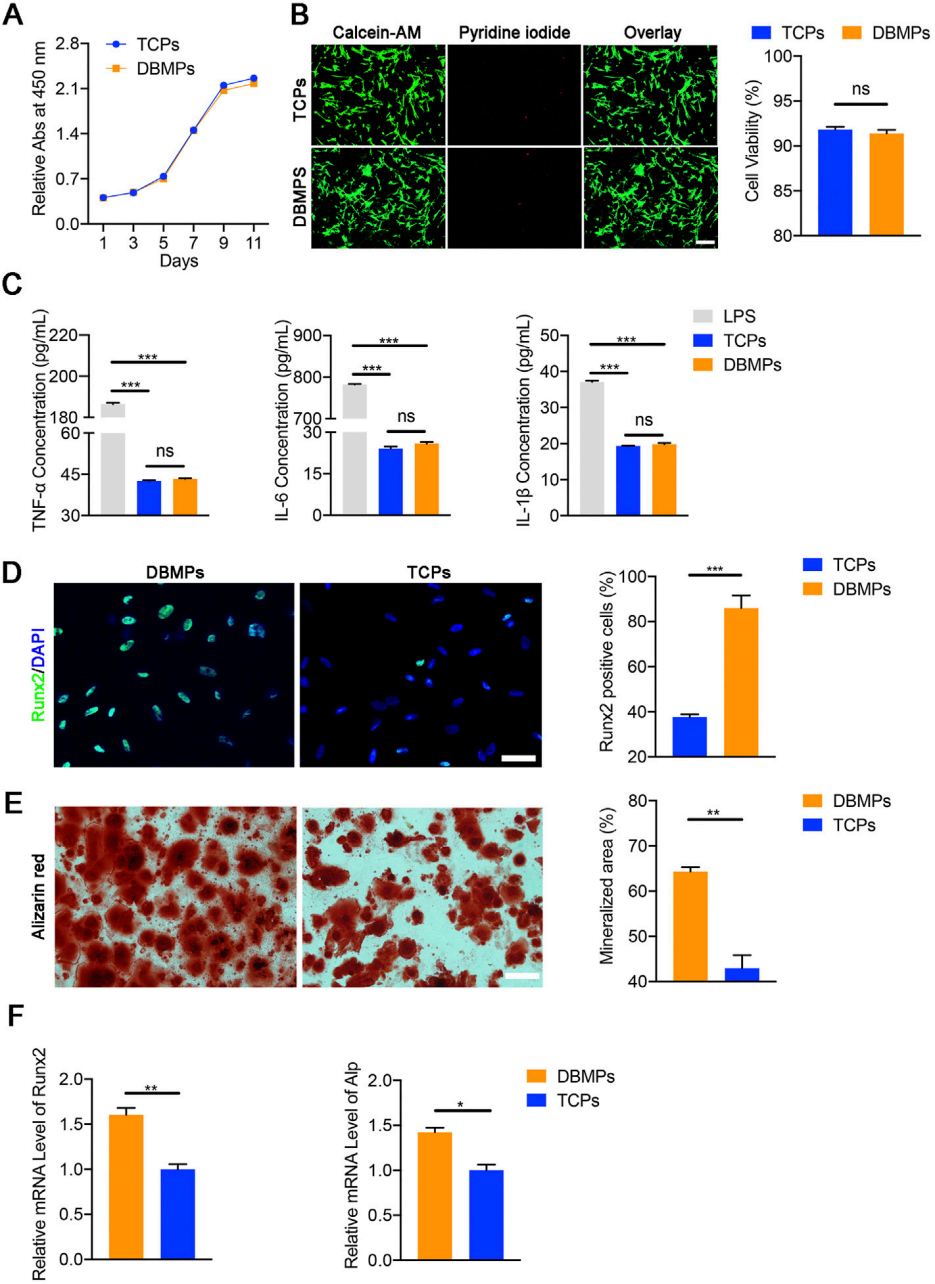
**(A)** Comparative cell proliferation assay of BMSCs seeded on the TCPs and DBMPs. **(B)** Live/dead cell analysis for the TCPs and DBMPs on which BMSCs had been seeded for 3 days. Representative images show the live (green) and dead (red) BMSCs in the TCPs and DBMPs and the viability analysis for the cells on the TCPs and DBMPs. Bar = 100 μm. **(C)** Pro-inflammatory cytokine (TNF-α, IL-6, and IL-1β) release by stimulation with TCPs, DBMPs, or LPS detected by ELISA assay. **(D)** Immunofluorescence staining of the Runx2 expression in BMSCs after culturing with TCPs or DBMPs. Comparative analysis of Runx2-positive cells after BMSCs were cultured on TCPs or DBMPs. Bar = 15 μm. **(E)** Alizarin red staining of BMSCs after culturing with TCPs or DBMPs. Comparative analysis of the alizarin red staining area after BMSCs cultured on TCPs or DBMPs. Bar = 20 μm. **(F)** qRT-PCR analysis shows that the expression of osteogenic (Runx2 and Alp) genes after BMSCs cocultured on TCPs or DBMPs. *n* = 4 for each group. Data are showed as means ± standard deviation (*p < 0.05, **p < 0.01, and ***p < 0.001).

As shown in Figure 3A, the BMSCs seeded on DBMPs showed a similar proliferation when compared with the BMSCs cultured on TCPs using a CCK8 assay. Additionally, using a live/dead assay, most of the BMSCs cocultured with DBMPs displayed green fluorescence (live cells) after 3 days of culture, while a few BMSCs presented red fluorescence (dead cells). Statistically, the viability of BMSCs on DBMPs was lower than that on TCPs, but the difference was not significant (Figure 3B).

### Immunogenicity of DBMPs

After RAW 264.7 cells were cultured with TCPS, DBMPs, and LPS, the supernatants of the TCP and DBMPs groups presented similar contents of pro-inflammatory cytokines (TNF-a, IL-6, and IL-1β). The supernatants of RAW 264.7 cells cultured in the TCP or DBMPs showed significantly lower expression levels than those under LPS stimulation (Figure 3C). These results indicated that DBMPs are biomaterials with low immunogenicity.

### Osteogenic Inducibility of DBMPs

At day 7 after seeding, significantly more BMSCs cocultured with the DBMPs expressed the osteogenic marker (Runx‐2) in comparison with the BMSCs cultured on the TCPS (Figure 3D). Meanwhile, qRT‐PCR results indicated that the expressions of Runx‐2 and Alp in the BMSCs cocultured with the DBMPs were significantly higher than those of BMSCs cultured on TCPs (Figure 3E). In addition, the BMSCs cocultured with DBMPs showed significantly greater positive staining area of alizarin red than the BMSCs cultured on the TCPS (Figure 3F).

### Appearance of Bone-forming Units and Their Injection

**FIGURE 4:**
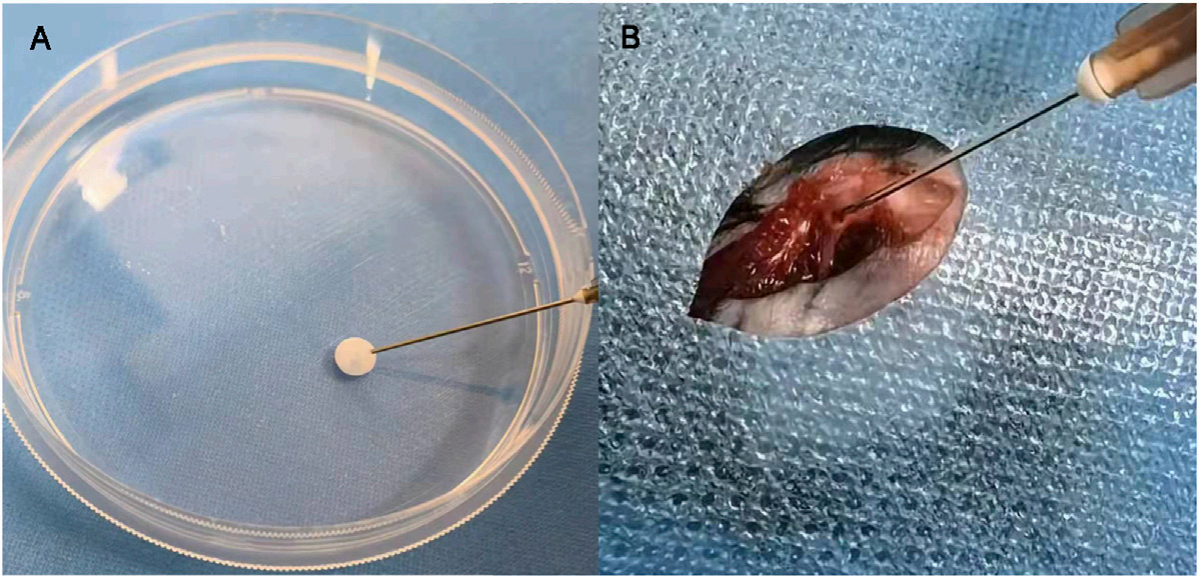
Bone-forming units can be delivered by fibrin glue **(A)**; the injection process of the bone-forming units *in vivo* **(B)**.

**FIGURE 5:**
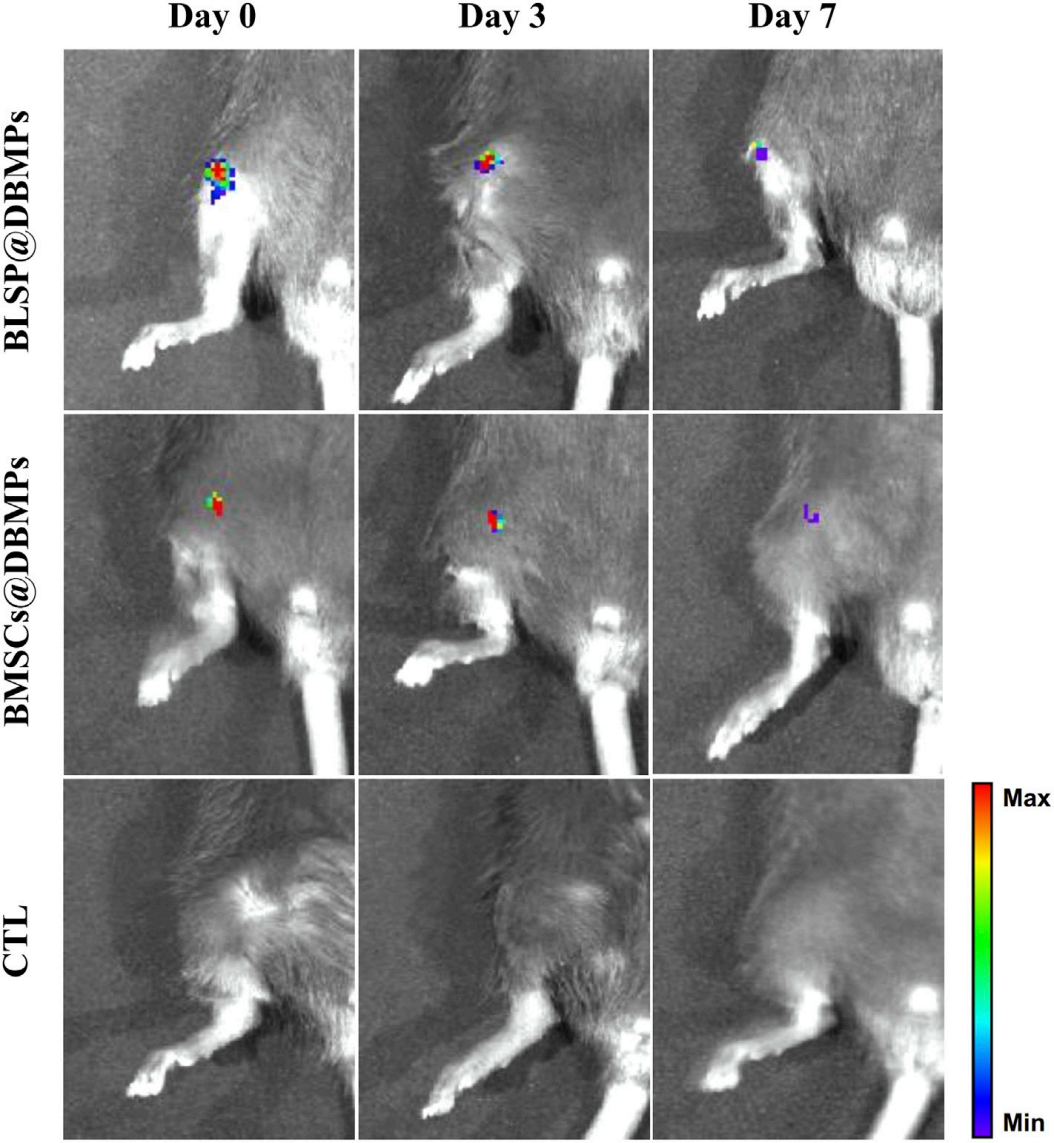
Gross morphology and IVIS analysis showed similar Dil intensity which was detected at the distal femur in the BMSCs@DBMP or BLSP@DBMP group and can last at least 7 days.

After the DBMPs, BMSCs@DBMPs or BLSP@DBMPs were mixed with the fiber glue; the mixture was transferred to a 1 ml syringe so that the hydrogel could be injected (Figure 4A). The injection-operation of DBMPs, BMSCs@DBMPs, or BLSP@DBMPs is shown in Figure 4B, indicating that the DBMPs, BMSCs@DBMPs, or BLSP@DBMPs could be delivered *via* injection to improve its handling. BLSPs or BMSCs at the DBMPs labeled with Dil before injection were used for tracking their fate and location. On the 3rd or 7th day after injection (Figure 5), IVIS images showed that a similar signal intensity of positive Dil was detected at the distal femur in the BMSCs@DBMP or BLSP@DBMP group. Although the signal intensity of positive Dil was gradually decreased from postoperative day 0 to postoperative day 7 in both BMSCs@DBMP group and BLSP@DBMP groups, a positive Dil area still exists at the distal femur at postoperative day 7. These results indicated that the BLSPs or BMSCs at the DBMPs remained viable and stayed at the defect site on day 7 after injection.

### *In Vivo* Performance of BLSP@DBMPs on Bone Defect Healing

**FIGURE 6:**
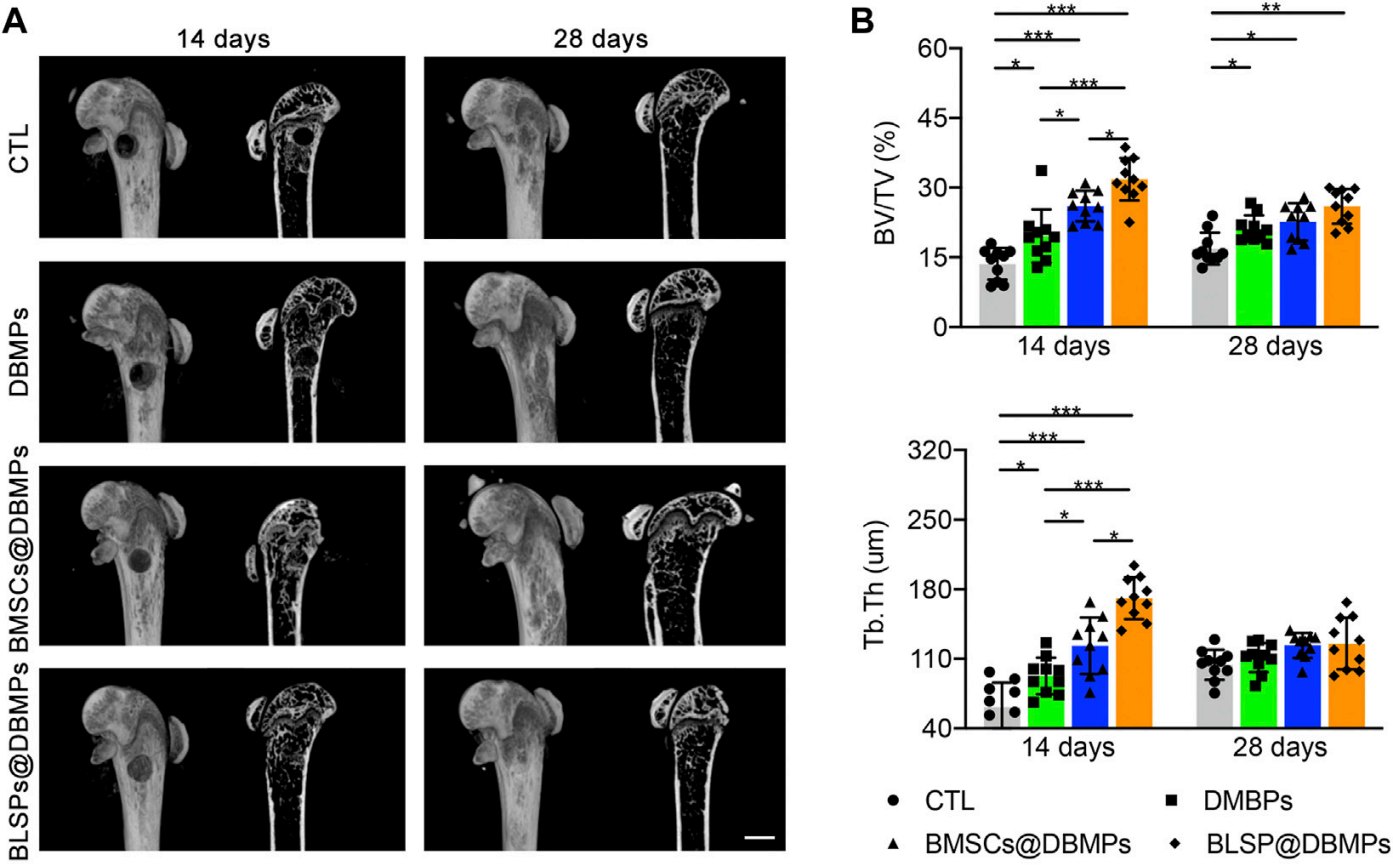
**(A)** Representative micro-CT images of the defected femur in the CTL, DBMP, BMSCs@DBMP, and BLSP@DBMP groups at postoperative 14 and 28 days. Bar = 1 mm. **(B)** Comparison of the BV/TV and Tb.Th in the newly formed bone among the four groups at different time points. *n* = 10 for each group. Data are showed as means ± standard deviation (*p < 0.05, **p < 0.01, and ***p < 0.001).

Micro-CT evaluation: the 3D reconstruction of the defect area clearly depicted the effects of different treatments on bone regeneration (Figure 6A). At postoperative 14 days, the bone defect in the BMSCs@DBMP group or BLSP@DBMP group was vague, but the density of the defect site in the BMSCs@DBMP group was the highest among the four groups. Statistically, the BLSP@DBMP group showed a significantly higher value of BV/TV and Tb.Th than these bone morphological parameters of other groups (Figure 6B). At postoperative 28 days, the defect site was filled with newly formed bone, and the defect was hard to observe in all groups. From the mid-sagittal tomography, the remodeling of the newly formed bone in the BLSP@DBMP group was nearly completed. Quantitatively, the BV/TV and Tb.Th of the newly formed bone at the defect site in the DBMP, BMSCs@DBMP, and BLSP@DBMP groups were higher than those of the CTL group at postoperative 28 days, while the BLSP@DBMP group showed the highest value among the three groups in these bone parameters (Figure 6B).

**FIGURE 7:**
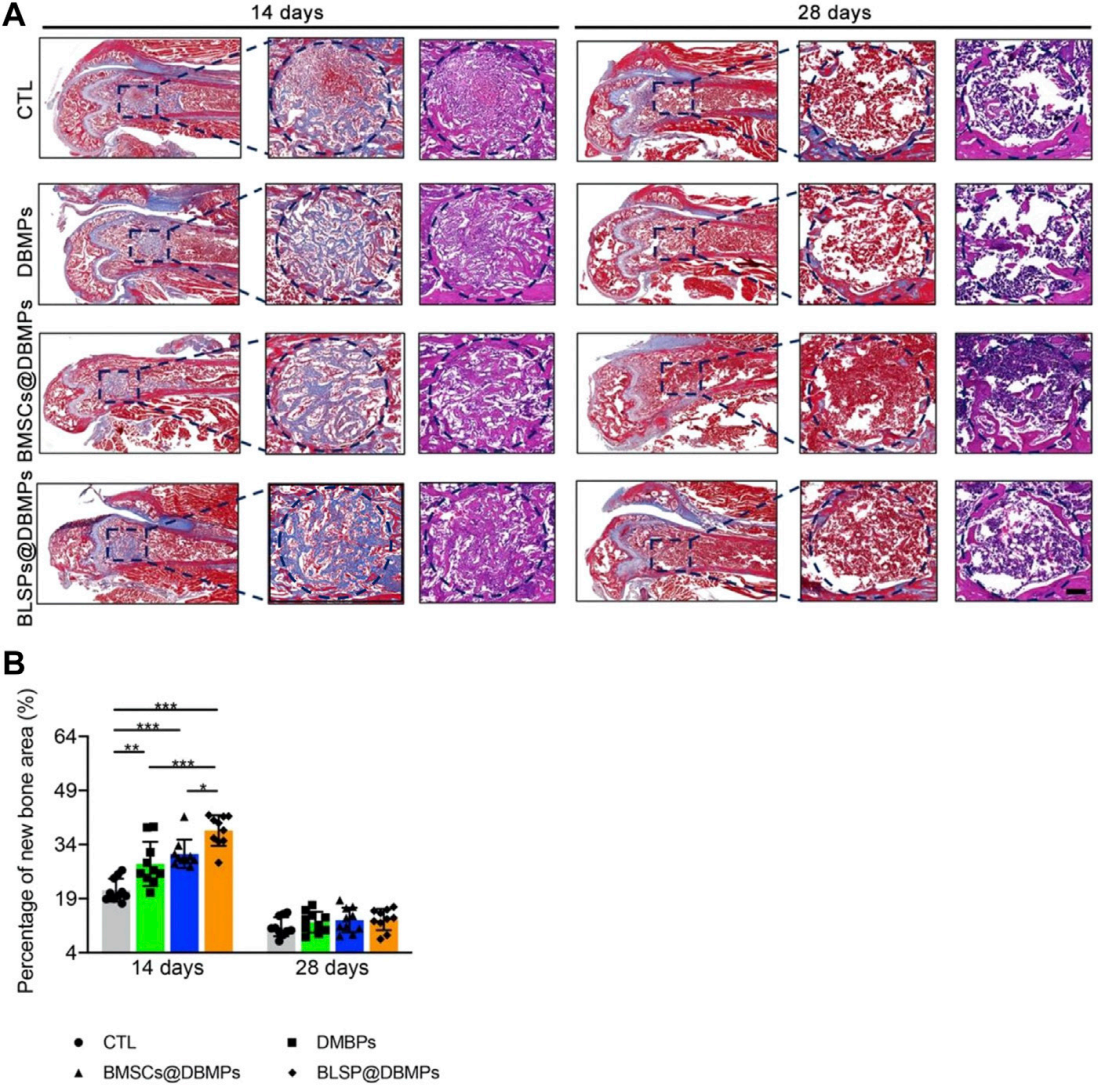
Histological analyses of regenerated bone at the defect site. **(A)** Representative images of the newly formed bone at the defect site in the sagittal view at postoperative day 14 or 28. The blue dotted circle indicates the defect site. Bar = 100 μm. **(B)** Percentage of the new bone area in the repaired bone defect area of each group at postoperative day 14 or 28. *n* = 10 for each group. Data are showed as means ± standard deviation (*p < 0.05, **p < 0.01, and ***p < 0.001).

**FIGURE 8:**
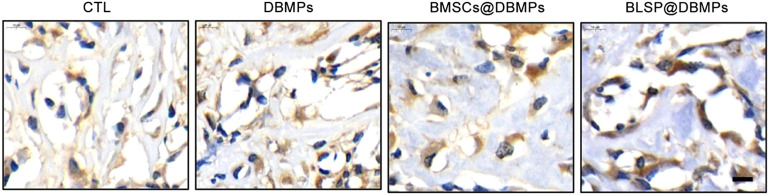
Immunohistochemical staining of Ocn in the repaired bone defect area of each group at postoperative day 14.

Histological evaluation: at postoperative 14 days, histological images show bone regeneration in the defect site for different treatments, as characterized by newly formed woven bone bridging the defect site (Figure 7A). The BMSCs@DBMP group and BLSP@DBMP group showed denser woven bone than the CTL group and DBMP group in the defect site. Compared with the DBMP group and BMSCs@DBMP group, BLSP@DBMP group presented the strongest effect on bone regeneration, as indicated by significantly larger new bone area (Figure 7B). Moreover, in the defect site, more Ocn positive cells were located at the new bone area in the BLSP@DBMP group when compared with the other groups (Figure 8). At postoperative 28 days, the newly formed woven bone in the defect site gradually remodeled into lamellar bone in all groups (Figure 7A). Statistically, the new bone area in the four groups was similar without significant difference (Figure 7B).

## Discussion

**FIGURE 9:**
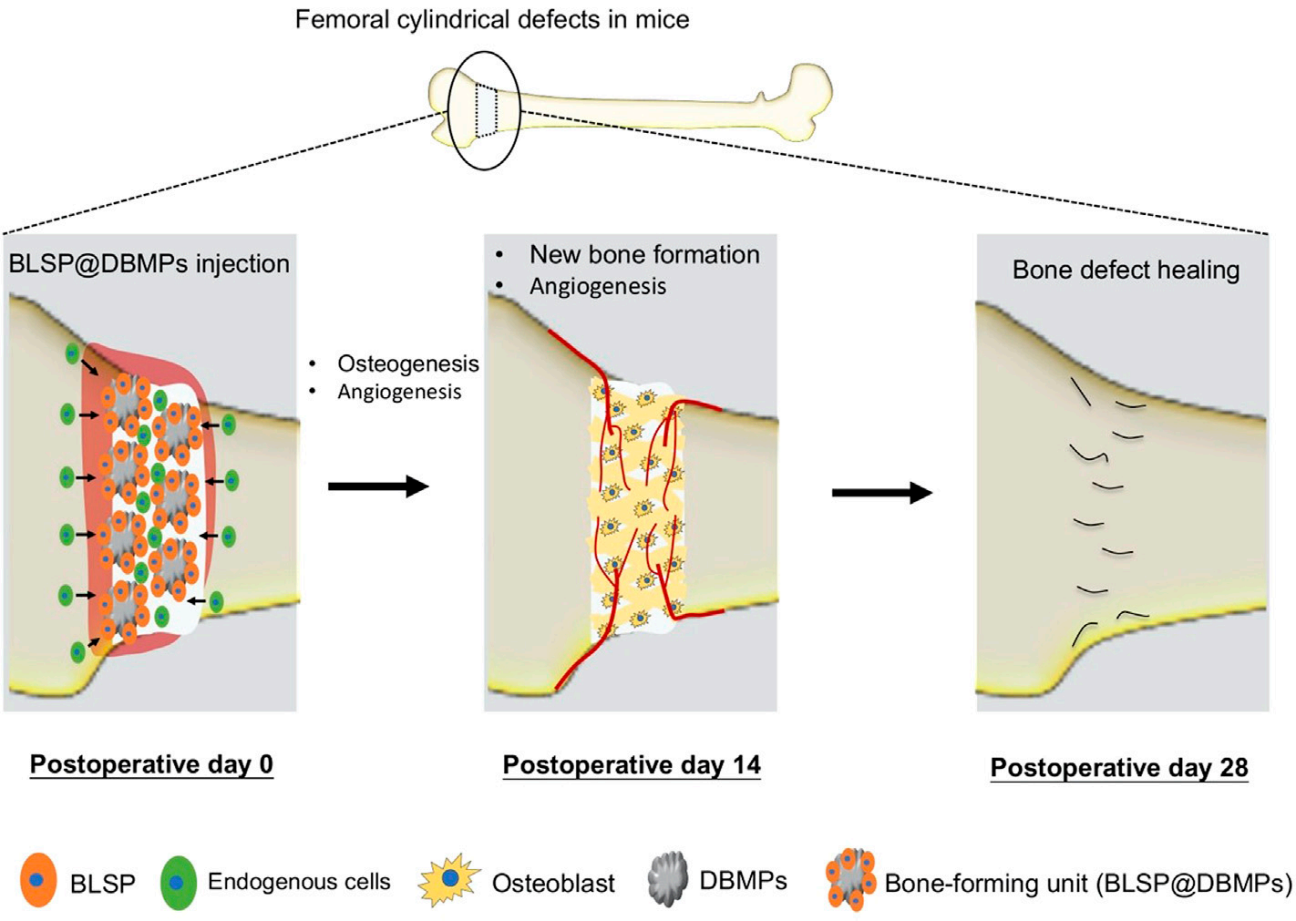
Schematic illustration of the application of the bone-forming units in bone defect healing and their potential mechanisms. The implanted BLSP attaching to the DBMPs secrete a series of bioactive substances stimulating angiogenesis at the bone defect site, and the DBMPs in the bone-forming units stimulate the attaching BLSP down into the osteogenic lineage, thus promoting bone defect healing.

Improving the repair of large segmental bone defects remains a difficult problem in orthopedic clinical treatment (Dumic-Cule et al., 2015). When the bone defect exceeds the critical size, self-regeneration is impossible (Schemitsch, 2017). Currently, autografts or allografts are widely used to fill the bone defects, and high performance is obtained in treating bone defects in clinical settings (Safdari et al., 2021). However, these strategies are limited by donor-site morbidity, infection, immune response, and pathogen transmission (Myeroff and Archdeacon, 2011; Migliorini et al., 2021). The development of bone TE presents promises in repairing large segmental bone defects (Kim et al., 2017). In this study, we established a protocol to construct a bone-forming unit by loading a subpopulation of osteoprogenitors with high osteogenic potential on the DBMPs and then injected the bioactive units into the defect site to improve the repair of bone defects. Our results indicated that the DBMPs are highly biomimetic to native bone in histology, microstructure, and ingredients, which function as a biomimetic scaffold for these seeded osteoprogenitors, and then stimulate the interacted osteoprogenitors differentiating into osteogenic lineage, thus making this bone-forming unit capable of enhancing the bone formation at the defect site (Figure 9). On that basis, we proposed the use of DBMPs and osteoprogenitors for constructing bone-forming units to improve the repair of large segmental bone defects.

During past years, tissue engineering provides us a promising treatment strategy for bone repair, utilizing a combination of biomaterial scaffolds, seeding cells, and growth factors (GFs) (Kim et al., 2017; Roseti et al., 2017; Moradi et al., 2018). Among the three factors, the biomaterial scaffolds can be fundamentally critical, which provides structural support and signals to regulate cellular responses for bone regeneration (Park et al., 2018). Currently, various studies have determined that a whole block of DBM was a promising biomaterial for bone regeneration and may be an ideal graft in clinical applications (Lee et al., 2016; Chen and Lv, 2018). Regrettably, a whole block of DBM shows a compact structure in organic and inorganic matrices, limiting cells migrating into the matrix to regenerate bone. To this shortcoming, we processed a whole block of DBM into powder. These DBMPs preserved the biomimetic properties and osteogenic inducibility of the whole block of DBM. Moreover, the crucial advantage of DBMPs originates from its ability to emulate the innate microenvironment for *in vivo* cellular homeostasis by providing space for cell anchorage and regulating cellular survival, proliferation, and differentiation *via* its proteins. Additionally, the powder form of DBM exposed more collagen to the interacted cells, convenient for organized biomineralization within and around collagen fibrils. Last, in comparison to the whole block of DBM, injectable bone-forming units can fill defects of any size or shape without requiring additional surgical procedures (Kim et al., 2017). These advantages of DBMPs make it an outstanding candidate biomaterial for bone regeneration. Generally, bone TE biomaterials should have suitable mechanical load while promoting bone regeneration (Biggemann et al., 2018; Zhao et al., 2021). However, the DBMPs fabricated in this study were injected with attaching cells using fibrin glue; thus, its mechanical properties are lower than those of the bone tissue. Fortunately, this study confirmed that our strategy was sufficient to support the repair of bone defects in a mouse model. As for clinical application, we think our injectable bone-forming units could act as a supplemental strategy for intramedullary nails or plates to enhance bone repair.

Apart from biomaterial scaffold, seeding cells is another critical component for tissue engineering (Yin et al., 2013; Font Tellado et al., 2015). In the past decades, the gold standard for stem cell isolation is based on its properties of adhering to plastic and forming colonies of fibroblast-like cells, described as mesenchymal stem cells (MSCs) (Caplan, 1991; Pittenger et al., 1999; Zhu et al., 2010). Several groups have further identified the MSCs by flow cytometry analysis using specific surface markers together with evaluating *in vitro* osteogenic, chondrogenic, and adipogenic differentiations (Méndez-Ferrer et al., 2010; Zhou et al., 2014). However, the MSCs isolated by the traditional way belong to a multicellular type mixture; individual MSCs vary in self-renewal capacity and multipotency. Thus, there is urgent need to prospectively isolate stem cells with higher purity (Phinney, 2012; Chan et al., 2015). Moreover, the lineage trajectory from MSCs to downstream differentiated cells has not been well characterized, and this has, thus, limited studies on stem cells, progenitors, and progeny responses in normal and diseased contexts. In our study, we used a comprehensive protocol described in a published literature to isolate stem cells, which begins with a mechanical and chemical digestion of bone that facilitates flow-cytometric sorting based on the expression of multiple cell surface markers (Gulati et al., 2018). Our results determined that the isolated osteoprogenitors are capable of self-renewal and osteogenic differentiation and have many advantages over the traditional BMSCs isolated by artificial plastic adhesion. This osteoprogenitor is freshly isolated by flow cytometry, which is purer and more representative of the endogenous cell types. More importantly, our osteoprogenitors showed higher osteogenic potential under osteogenic induction when compared with the transitional BMSCs. Thus, these osteoprogenitors were isolated and selected as seeding cells for preparing bone-forming units in our study.

Using the femoral epiphysis bone defect in a mouse model, quantification of the static parameters of bone formation by micro-CT analysis shows that the BV/TV and Tb.Th of the new bone at the defect site in the DBMP, BMSCs@DBMP, and BLSP@DBMP groups were higher than those of the CTL group at postoperative day 14, while the BLSP@DBMP group showed the highest value in these bone parameters among the three groups. Histological results also showed that the BLSP@DBMP group had the best bone formation ability compared with the other three groups. The reasons for this result may be that: 1) DBMPs acted as scaffold for cell adhesion. When the DBMPs are injected into the bone defect site, various endogenous cells will migrate into these DBMPs. Under the stimulation of DBMPs, these endogenous cells differentiate into osteogenic lineage while gradually internalizing these DBMPs to form bone tissue. However, this process is complicated and slow; thus, the DBMP group showed limited bone formation at postoperative day 14. With the passage of time, these DBMPs on promoting bone defect healing gradually function, leading to significant better bone formation at postoperative 28 days. 2) TE strategies based on the combination of BMSCs and scaffold have been reported to show good performance in bone regeneration (Derubeis and Cancedda, 2004; Luby et al., 2019). Similarly, we used the fibrin glue to deliver BMSCs@DBMPs into the defect site to enhance bone regeneration. Regrettably, the bone defect treated with BMSCs@DBMPs was not healed satisfactorily as we expected. This may be caused by the BMSCs isolated by the conventional method which has a limited osteogenic potential. When the BLSP/DBMPs were applied, we found that the bone defect was healed significantly better than the BMSCs@DBMPs or DBMPs only at postoperative day 14. 3) In the BLSP/DBMP group, the BLSP was attached on the DBMPs, which provided a support for BLSP. After being injected into the defect site, the attached BLSP secreted a broad spectrum of exosomes or biomolecules to optimize the local immune environment, thus promoting angiogenesis and stromal cell migration at this site. On the other hand, the injected cells directly differentiated into osteogenic lineage under the stimulation of DBMPs. Hence, more new bones were formed at the defect site of BLSP/DBMPs in comparison with other groups. To prove these interpretations, more experiments should be performed.

There are several limitations to our study. First, although this study determined that the BLSP/DBMPs show superior performance on bone defect healing, we did not explore the specific signaling pathway of the DBMPs promoting the osteogenic potential of BLSPs. Second, the isolation and culture procedures of BLSPs are time-consuming, which may be inconvenient for clinical application. Hence, developing a method to shorten the isolation and culture procedures of BLSP is urgent and meaningful. Additionally, the gating strategy used to sort the BLSP from human bones was different from that of the mouse bones (Chan et al., 2018); more studies should be performed before clinical application. Third, the observation time point of the animal experiment section was single. It was not explored in depth whether the injected BLSPs were directly differentiated into osteocytes for bone defect healing or they were functioned by the production of related cytokines. Fourth, only radiological and histological evaluations of the regenerative capability of the BLSP/DBMPs were carried out in a mouse model; mechanical behavior of the healed bone should also be evaluated in future studies. Finally, this injectable bone-forming unit had insufficient mechanical properties; thus, it can only be used for treating bone defects in non-heavy environments or bone defects after internal fixation.

In this work, an injectable bone-forming unit (BLSP/DBMPs) was constructed by loading a subpopulation of BLSPs with superior osteogenic potential on the DBMPs, which not only improved the handling of the DBMPs and BLSPs but also contributed to maintaining the viability and differentiation capability of the BLSP. Additionally, this injectable bone-forming unit is beneficial for enhancing bone defect healing in a mouse femoral epiphysis bone defect model. This study indicates that this injectable bone-forming unit has good clinical transformation and application potential for bone repair in the future.

## Data Availability

The original contributions presented in the study are included in the article/Supplementary Material, further inquiries can be directed to the corresponding authors.
